# Anti-Mesothelin CAR T cell therapy for malignant mesothelioma

**DOI:** 10.1186/s40364-021-00264-1

**Published:** 2021-02-15

**Authors:** Laura Castelletti, Dannel Yeo, Nico van Zandwijk, John E. J. Rasko

**Affiliations:** 1 Li Ka Shing Cell & Gene Therapy Program, The University of Sydney, Camperdown, Australia; 2 Faculty of Medicine and Health, The University of Sydney, Camperdown, Australia; 3 Cell and Molecular Therapies, Royal Prince Alfred Hospital, Sydney Local Health District (SLHD), Camperdown, Australia; 4 Concord Repatriation General Hospital, Sydney Local Health District (SLHD), Concord, Australia; 5 Gene and Stem Cell Therapy Program Centenary Institute, The University of Sydney, Camperdown, Australia

**Keywords:** Cancer, Malignant mesothelioma, Malignant pleural mesothelioma, Mesothelin, CAR T cells, Tumor microenvironment, Immunotherapy

## Abstract

Malignant mesothelioma (MM) is a treatment-resistant tumor originating in the mesothelial lining of the pleura or the abdominal cavity with very limited treatment options. More effective therapeutic approaches are urgently needed to improve the poor prognosis of MM patients. Chimeric Antigen Receptor (CAR) T cell therapy has emerged as a novel potential treatment for this incurable solid tumor. The tumor-associated antigen mesothelin (MSLN) is an attractive target for cell therapy in MM, as this antigen is expressed at high levels in the diseased pleura or peritoneum in the majority of MM patients and not (or very modestly) present in healthy tissues. Clinical trials using anti-MSLN CAR T cells in MM have shown that this potential therapeutic is relatively safe. However, efficacy remains modest, likely due to the MM tumor microenvironment (TME), which creates strong immunosuppressive conditions and thus reduces anti-MSLN CAR T cell tumor infiltration, efficacy and persistence. Various approaches to overcome these challenges are reviewed here. They include local (intratumoral) delivery of anti-MSLN CAR T cells, improved CAR design and co-stimulation, and measures to avoid T cell exhaustion. Combination therapies with checkpoint inhibitors as well as oncolytic viruses are also discussed. Preclinical studies have confirmed that increased efficacy of anti-MSLN CAR T cells is within reach and offer hope that this form of cellular immunotherapy may soon improve the prognosis of MM patients.

## Introduction

Malignant mesothelioma (MM) is an aggressive, treatment-resistant and relatively rare cancer affecting the mesothelial lining of the pleura or abdominal cavity, often elicited by prior asbestos exposure [1, 2]. There are three histological subtypes of MM: epithelioid, sarcomatoid and biphasic. The epithelioid form of MM is the most common subtype and has a better prognosis than the biphasic and sarcomatoid types [3, 4]. MM has shown resistance against traditional oncological therapies and one of the lowest survival rates of any cancer type. Novel immunotherapeutic approaches targeting the tumor-associated antigen (TAA) mesothelin (MSLN) are currently being tested in clinical trials and have the potential to improve survival rates and prognosis in MM [5, 6], especially anti-MSLN Chimeric Antigen Receptor (CAR) T cell therapy, which is the focus of this review.

**Fig. 1:**
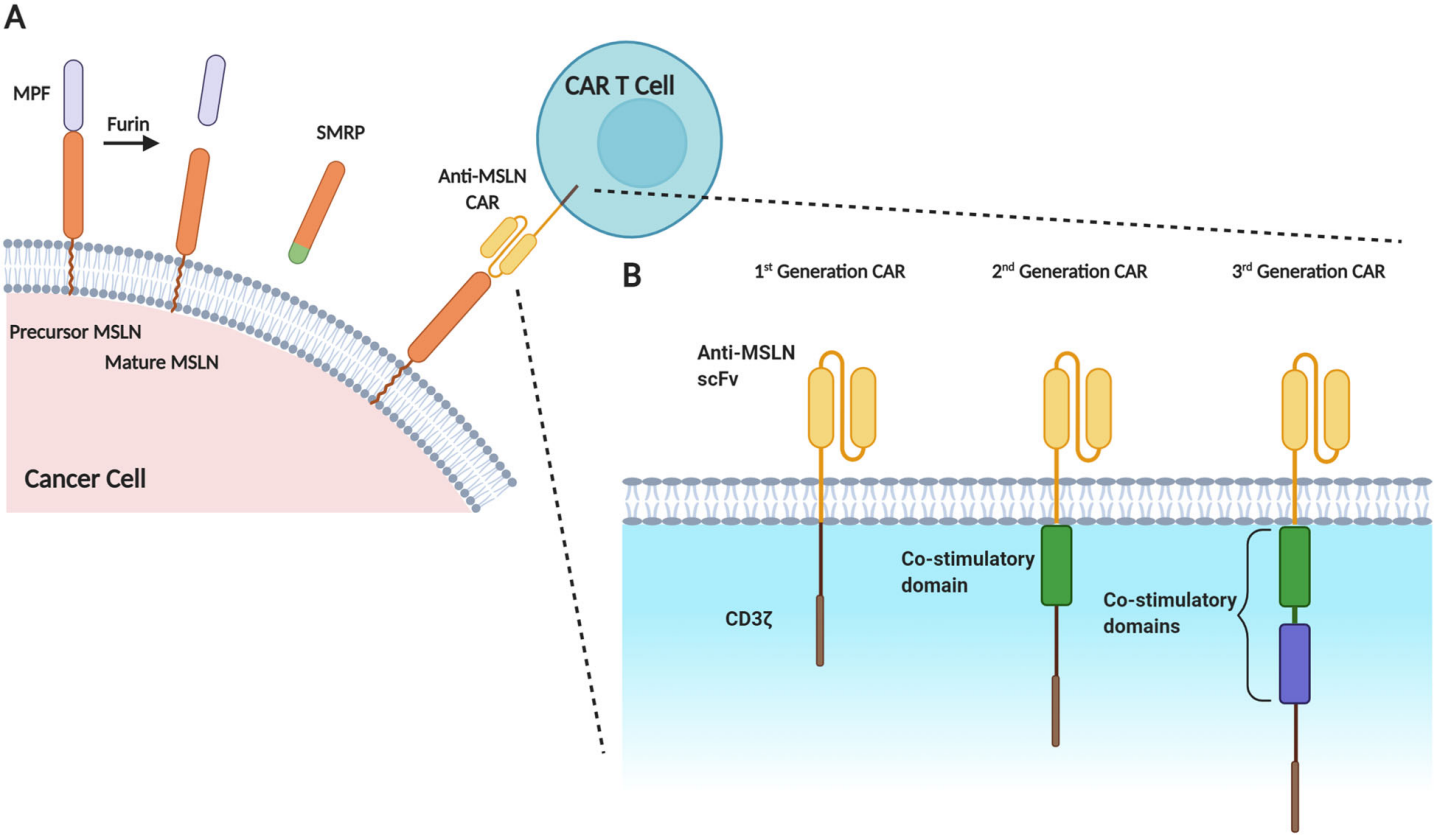
**a** Maturation of Mesothelin (MSLN). MSLN is initially expressed on the membrane of cancer cells as a 71 kDa precursor protein which is cleaved by the endoprotease Furin to release the 31 kDa Megakaryocyte Potentiating Factor (MPF). The mature 40 kDa-MSLN remains bound to the cell membrane via a glycophosphatidylinositol (GPI) anchor. Surface MSLN can also be released from the GPI anchor by proteases, resulting in a soluble form of MSLN (soluble MSLN-related peptide - SMRP). **b** Anti-MSLN CAR Generations. The scFv of MSLN CAR T cells binds membrane-bound MSLN and the signaling depends on the CAR generation used. The endodomain of first generation CARs contains CD3ζ only; in second generation CARs, there is the addition of a single costimulatory domain (often CD28 or 4–1-BB); and in third generation CARs, two costimulatory domains are incorporated

MSLN was first discovered in 1992 in an effort to find new surface targets for monoclonal antibody immunotherapy [7]. It is expressed at low levels in healthy mesothelial cells of the pleura, pericardium and peritoneum, whereas virtually every MM case exhibits significant MSLN expression in the tumor biopsy [8]. The physiological role of MSLN in healthy tissues is unclear and is likely to be non-essential [9]. MSLN is initially expressed as a precursor protein of 71 kDa which is then cleaved by Furin causing a 31 kDa protein called megakaryocyte potentiating factor (MPF) to be shed, while the remaining 40 kDa fragment, MSLN, stays bound to the cell membrane through a glycosylphosphatidylinositol (GPI) anchor [7]. Surface MSLN can also be shed creating soluble mesothelin-related peptide (SMRP) which can be detected in the blood of MM patients [10] (Fig. 1a).

MSLN has been the focus of immunotherapy research since its discovery. The features making MSLN an ideal immunotherapeutic target in MM are: A) the high level of MSLN expression in cancer tissue and low-to-no expression in healthy tissue, thus reducing possible ‘on target/off-tumor’ toxicities [11]; B) 85–90% of cases in the epithelioid subtype of MM present with high expression of MSLN [12]; and C), its expression at high levels has been associated with increased aggressiveness and invasiveness [13].

The extracellular domain of MSLN comprises three contiguous elements: region I (residues 296–390), II (391–486), and III (487–598) [14]. Region I is the membrane-distal region and can bind to the mucin MUC16 (also known as CA125), which is also expressed by the majority of MM cells and associated with cancer aggressiveness. This MSLN-MUC16 interaction has been shown to be important for tumor cell adhesion and metastasis [15, 16] and is the main target of existing anti-MSLN immunotherapies [5, 12, 17–20], including anti-MSLN CAR T cell therapy.

CARs are engineered proteins expressed on the surface of T cells. Their structure has evolved since the 1990s, building on successful CD19-based CAR T cell therapies [21]. Typical features include the ectodomain, containing the single-chain variable fragment (scFv) identifying and binding to a specific TAA; a hinge; a transmembrane domain; and an endodomain, which contains the signaling domains. The evolving endodomain structures gave rise to different CAR generations (Fig. 1b) [22]. As new challenges to CAR T cell function are discovered, new costimulatory domains and measures to resist the immunosuppressive tumor microenvironment (TME) are being explored.

Autologous anti-MSLN CAR T cell therapy represents a new paradigm in MM immunotherapy. Compared to other MSLN-targeted therapies for MM, it offers the potential to promote immune surveillance and avoid tumor recurrence through CAR T cell persistence in the patients’ body and reactivation after further antigen encounter [23]. Anti-MSLN CAR T cells have also been shown not to react with SMRP [24] and only activate their cytotoxic activity against membrane-bound MSLN [25, 26].

Another characteristic that makes CAR T cells an attractive therapy against MM is the synergy with checkpoint inhibitors (CI) [27]. CI target receptors such as programmed cell death protein-1 (PD-1) and cytotoxic T lymphocyte antigen 4 (CTLA-4), which are expressed on T cells after activation and interaction with stimuli in the TME. Upon binding to their ligands (e.g. programmed cell death ligand, PD-L1), these molecules mediate an exhausted phenotype and apoptosis, thus limiting T cell anti-tumor activity [28, 29]. Following the success of a phase III trial using CI showing a 4-month survival gain [30], the FDA has recently approved the Nivolumab (against PD-1) and Ipilimumab (against CTLA-4) combination for unresectable MM. Itis expected that this combination immunotherapy will replace the standard of care chemotherapy pemetrexed/cisplatin combination [31]. Anti-MSLN CAR T cell therapy is expected to work well in combination with CI immunotherapy as it could help to resist exhaustion and increase persistence and efficacy of CAR T cells.

This review summarizes the results of recent clinical trials using anti-MSLN CAR T cell therapy against MM. We also highlight the progress made in anti-MSLN CAR T cell engineering which resulted in increased infiltration, persistence and anti-tumor activity.

## Clinical trials using anti-Mesothelin CAR T cell therapy in mesothelioma

Recent anti-MSLN CAR T cell clinical trials against MM are summarized in Table 1. They highlight the variety of strategies for anti-MSLN CAR T cell production and genetic engineering.

**Table 1:** Anti-mesothelin CAR T cell therapy clinical trials in malignant mesothelioma

clinicaltrials.gov Identifier	Phase	Intervention	Additional therapy	Cancer type	Delivery	Sponsor	Study completion	Construct information	Status / Outcome
NCT01583686	I/II	Anti-mesothelinCAR-transduced peripheral blood lymphocytes	fludarabine, cyclophosphamide, IL-2	MSLN expressing tumors	Systemic	National Cancer Institute (NCI), USA	Dec-18	SS1 scFv	Terminated due to slow/insufficient accrual. Positive results for safety but low efficacy. Best OR: 1/15 with SD. 14/15 with PD.
NCT01355965	I	mRNA anti-MSLN CAR-T cells		MPM, PDAC	Systemic	University of Pennsylvania, USA	Oct-15	SS1 mouse scFv fused to the 4–1-BB and CD3ζ signaling domains	Completed. Positive results (Primary endpoint: safety). Best OR: 2/18 patient showed transient response (Ref. 32, 33).
NCT02159716	I	Anti-MSLN CAR-T cells (CART-meso)		MPM, PDAC, ovarian	Systemic	University of Pennsylvania, USA	Nov-15	SS1 mouse scFV fused to the 4-1BB and CD3ζ signaling domains	Completed. Positive results (Primary endpoint: safety). Low persistence and low tumor infiltration were observed. Best OR: 6/15 patients with SD (Ref. 34).
NCT03054298	I	Anti-MSLN CAR-T cells (huCART-meso)	cyclophosphamide	MSLN expressing tumors	Systemic and intrapleural	University of Pennsylvania, USA	Mar-21	SS1 humanized scFV	Recruiting.
NCT03608618	I	mRNA anti-MSLN CAR PBMC (MCY-M11)	cyclophosphamide	Peritoneal Mesothelioma,Fallopian Tube and ovary Adenocarcinoma,Primary Peritoneal Carcinoma	Intraperitoneal	MaxCyte, Inc., USA	May-22	NA	Recruiting. Preliminary results (ASCO 2020): positive results on safety. 4/11 patients with SD (Ref. 35).
NCT02414269	I/II	Anti-MSLN CAR-T cells with suicide switch (iCasp9M28z)	cyclophosphamide or pembrolizumab	MPM	Intrapleural	Memorial Sloan Kettering Cancer Center, USA	Apr-21	anti-MSLN scFv linked to the CD28 and CD3ζ signaling domains plus suicide gene inducible caspase 9	Recruiting. Preliminary results (AACR 2019, ASCO 2019): positive results on safety, 2/19 patients with CR, 5/19 with PR, 4/19 with SD (Ref. 36, 37).
NCT04577326	I	anti-MSLN CAR T cells with intrinsic anti-PD1 inhibition (M28z1XXPD1DNR)	cyclophosphamide	MPM	Intrapleural	Memorial Sloan Kettering Cancer Center, USA	Sep-22	anti-MSLN scFv linked to the CD28 and CD3ζ signaling domains plus PD-1 DNR and 1XX ITAM modification	Recruiting.
NCT03907852	I/II	Anti-MSLN TRuC (TC-210)	cyclophosphamide, pembrolizumab, fludarabine	MSLN expressing tumors	Systemic	TCR^2^ Therapeutics, USA	Jan-23	single-domain anti-MSLN antibody fused to the CD3-ε subunit	Recruiting. Preliminary results in 5 patients show positive results on safety and 2/5 patients with unconfirmed PR (Ref. 38).
NCT04489862	I	Anti-MSLN CAR-T cells expressing PD-1 nanobodies		MPM, NSCLC	Systemic	Wuhan Union Hospital, China	Dec-22	NA	Recruiting.
NCT03638206	I/II	Anti-MSLN CAR-T cells	cyclophosphamide, fludarabine	MPM	Systemic	Shenzhen BinDeBio Ltd., China	Mar-23	NA	Recruiting.

Abbreviations: *MSLN* mesothelin, *MPM* malignant pleural mesothelioma, *PDAC* pancreatic ductal adenocarcinoma, *OR* overall response, *SD* stable disease, *PD* progressive disease, *PR* partial response, *CR* complete response, *PBMC* peripheral blood mononuclear cells, *PD-1* Programmed cell death protein 1, *KO* knock out, *NSCLC* non-small cell lung cancer, *DNR* Dominant negative receptor

A trial led by the National Cancer Institute used anti-MSLN CAR T cells carrying an SS1 scFv against MSLN expressing tumors, including MM (NCT01583686). Patients received a lymphodepleting regimen of fludarabine and cyclophosphamide before anti-MSLN CAR T cell infusion. Lymphodepletion is advised before cell therapy as it decreases the number of immunosuppressive regulatory T cells (T_regs_) as well as the competition from other lymphocytes thereby creating a more favorable environment for CAR T cells and potentially increasing CAR T cell persistence and efficacy [39, 40]. Patients were also given IL-2 which helps T cell expansion and differentiation upon antigen encounter [41]. This trial was terminated after 6 years due to insufficient patient accrual. The therapy showed a manageable safety profile, but efficacy was low, with only one out of 15 patients showing stable disease (SD).

**Fig. 2:**
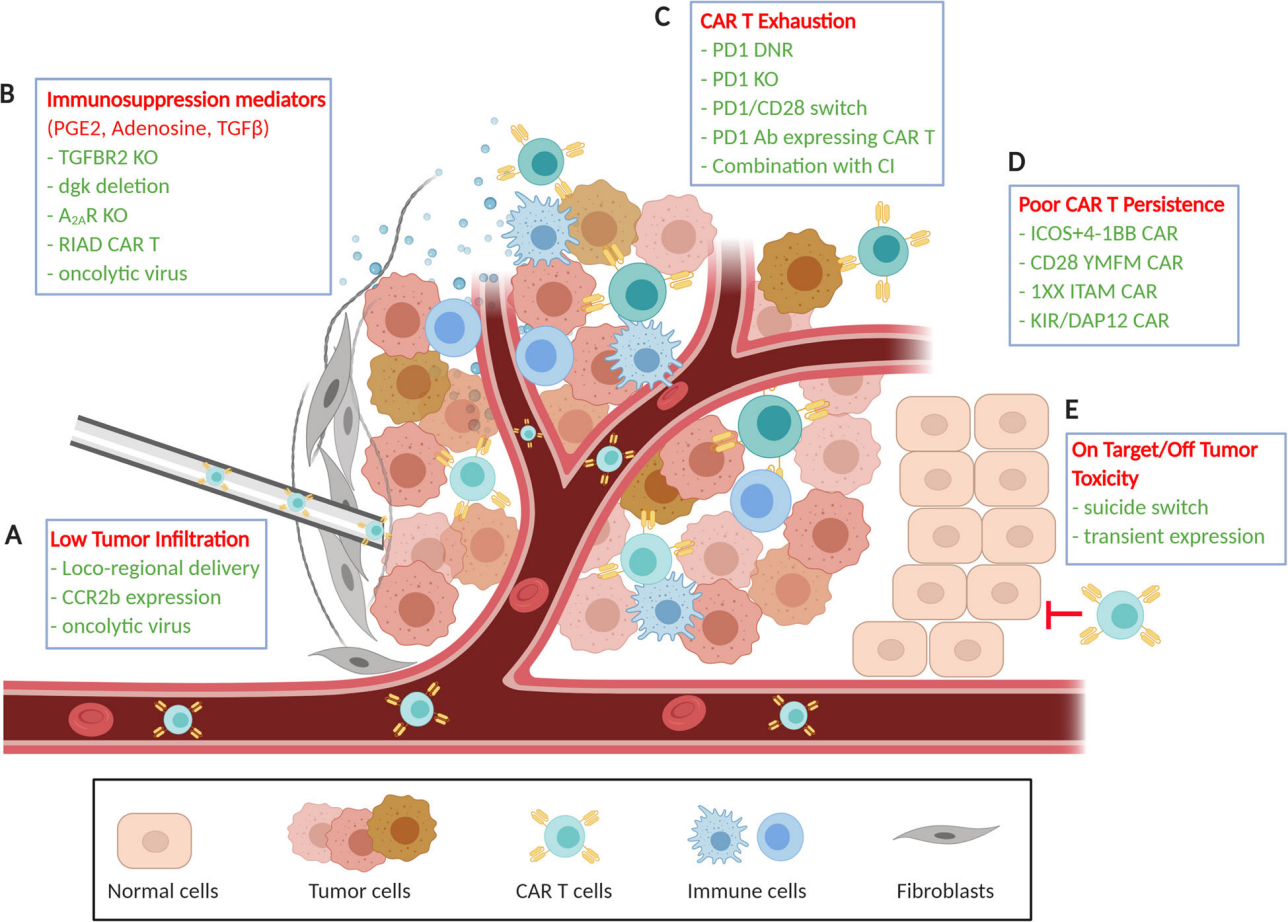
Barriers to MSLN CAR T cell activity in MM (in red) and strategies to overcome them (in green). **a** Low tumor infiltration caused by the physical barrier of the stroma in MM could be overcome by intrapleural or intraperitoneal delivery of anti-MSLN CAR T cells or by expressing the CCR2b chemokine receptor on anti-MSLN CAR T cells, which attracts them to the tumor. Combination with a vaccinia virus expressing CXCL11 may also increase anti-MSLN CAR T cell trafficking to the tumor. **b** Soluble immunosuppression mediators prostanglandin E (PGE2), adenosine and TGF-β contribute to the reduction of anti-tumor activity of anti-MSLN CAR T cells. Strategies that successfully rescued anti-MSLN CAR T cell anti-tumor activity include the knock out (KO) of receptors for TGFβ (TGFBR2) and adenosine (A_2A_R), the deletion of diacylglycerol kinase (dgk), and the insertion of a ‘regulatory subunit I anchoring disruptor’ (RIAD) in anti-MSLN CAR T cells. The combination of anti-MSLN CAR T cells with TGF-β-targeting oncolytic viruses and TNFα-IL2-producing oncolytic viruses has also helped enhance their efficacy. **c** Exhaustion due to PD-1/PD-L1 signaling can be counteracted via knock out of PD-1 via CRISPR/Cas9 or by inserting a PD-1 DNR or a PD-1/CD28 switch receptor. Engineering of CAR T cells to secrete anti-PD-1 antibodies has also been studied. **d** Anti-MSLN CAR T cells with improved CAR design have been shown to have increased persistence and efficacy in mouse models. **e** CAR T safety can be improved by limiting ‘on target/off tumor’ toxicities through the introduction of a suicide switch in anti-MSLN CAR T cells or the use of mRNA anti-MSLN CAR with transient expression

Based on promising results from their preclinical studies [25, 42], the University of Pennsylvania completed two phase I trials in MM, testing their second generation anti-MSLN CAR T cell therapy carrying a murine derived SS1 scFv targeting MSLN region I. In the first trial against malignant pleural mesothelioma (MPM) and pancreatic ductal adenocarcinoma (PDAC) (NCT01355965), T cells were transfected with mRNA to express the CAR transiently and thus limit potential ‘on target/off tumor’ toxicities (Fig. 2e). One MPM patient experienced a severe anaphylactic reaction after the third anti-MSLN CAR T cells infusion that was attributable to the formation of antibodies against the murine CAR. Repeated administration of the mRNA-anti-MSLN CAR is likely to have caused this reaction, thus raising concerns about the potential immunogenicity of murine-derived CAR when administered in recurrent doses [32]. Overall this trial showed a satisfactory safety profile and a transient tumor response was noted in 2 out of 18 patient [33]. These results prompted this group to stably transduce T cells via lentiviral vectors carrying the same CAR construct in their next trial (NCT02159716). Anti-MSLN CAR T cells were administered intravenously to 15 patients with MPM, PDAC and ovarian cancer in two different doses, as monotherapy or in combination with the lymphodepleting agent cyclophosphamide. Lymphodepletion increased CAR T cell expansion but not persistence. Moreover, low persistence and low tumor infiltration were observed, which may explain the limited clinical activity. The best overall response was stable disease (SD) in 11 out of 15 patients at day 28 post-infusion, of which 5 showed progressive disease (PD) at a later time point [34]. A third trial is currently recruiting and is testing a fully humanized anti-MSLN CAR T cell therapy administered intravenously or intrapleurally (NCT03054298). Regional intrapleural or intraperitoneal administration may help to increase efficacy and persistence as delivering cells directly into the tumor circumvents lack of trafficking and homing to the tumor due to the physical barrier of the stroma in MM (Fig. 2a).

Regional delivery was also employed in a phase I dose-escalation trial against ovarian cancer and peritoneal mesothelioma organized by MaxCyte (NCT03608618). Cells were injected via intraperitoneal infusion once a week for 3 weeks. Interestingly, a new manufacturing platform was implemented. Rather than transfecting T cells, this platform involved transiently transfecting fresh, non-expanded, autologous peripheral blood mononuclear cells (PBMC) with mRNA carrying the human anti-MSLN CAR (MCY-M11). This manufacturing process aims to rapidly produce CAR cells in a single day in order to achieve faster and lower cost therapeutics. This study is ongoing but preliminary results presented at ASCO 2020 showed that the treatment was well tolerated and 4 out of 11 patients showed initial SD, which was maintained in 3 patients for more than two months. The next part of the trial includes the addition of preconditioning chemotherapy and multiple cycles designed to increase efficacy [35].

Regional anti-MSLN CAR T delivery is also being used by the Sadelain team at Memorial Sloane Kettering Cancer Center (MSKCC) in a phase I/II trial against MM (NCT02414269), based on the success of the same approach in an orthotopic mouse model [26]. Anti-MSLN CAR T cells in this trial also carry a suicide switch based on the iCasp9 cell-suicide system [43], which can be activated in case of severe ‘on target/off tumor’ toxicities (Fig. 2e). One cohort of patients will also be treated with the anti-PD-1 CI pembrolizumab, to overcome CAR T cell exhaustion and prolong functional persistence. Promising preliminary results were presented at AACR 2019 and ASCO 2019, showing that this combination was well tolerated with no ‘on-target/off-tumor toxicities’. Anti-tumor activity was noted and the best responses among the 19 MPM patients (of which 13 received pembrolizumab) were two complete metabolic responders (PET scan confirmed). A further 5 patients had a partial response and 4 exhibited SD. These results suggest that synergy between CAR T cells and the anti-PD-1 therapy may have played a role [36, 37].

In an effort to improve persistence of CD28/CD3ζ CAR T cells, the same group at MSKCC successfully calibrated the signal strength on their CD28/CD3ζ anti-MSLN CAR T cells by mutating tyrosine residues on 2 of the 3 CD3ζ immunoreceptor tyrosine-based activation motifs (ITAM), thus blocking their phosphorylation and effectively creating a weaker downstream signal (1XX CAR). This CAR engineering resulted in increased persistence of highly functional CAR T cells [44]. Their anti-MSLN 1XX CAR T cells also carry PD-1 dominant-negative receptors (DNR), which act as decoy for PD-L1 molecules, thus decreasing CAR T cell exhaustion (M28z1XXPD1DNR CAR T cells or ATA2271) [45]. A phase I clinical trial has just started using these engineered anti-MSLN CAR T cells against MM (NCT04577326).

To avoid CAR T cell exhaustion, TCR^2^ Therapeutics is combining their anti-MSLN T cell therapy with a PD-1 inhibitor in a phase I/II trial against MSLN expressing tumors, including MM (NCT03907852). Interestingly, instead of a CAR, this study utilizes a proprietary T cell receptor (TCR) fusion construct called TRuC (TC-210). TC-210 is a single-domain anti-MSLN antibody fused to the CD3-ε subunit which, upon expression, is incorporated into the endogenous TCR complex (NCT03907852). TC-210 is able to recognize and bind to MSLN on cancer cells, thus stimulating the cytotoxic activity of T cells. Preliminary results from 5 patients (4 with MM and 1 with ovarian cancer) in this study showed a manageable toxicity profile as well as some tumor shrinkage. Enrolment in this study is ongoing and combination therapy with pembrolizumab is being tested [38].

A recently initiated trial in China uses augmented anti-MSLN CAR T cells that have been genetically modified to secrete anti-PD-1 nanobodies to avoid PD-1-mediated inhibitory signaling (NCT04489862).

The clinical trials illustrated here present a variety of strategies of anti-MSLN T cell therapy in terms of CAR construct, transduction method, combination therapy, and delivery method. Different signaling domains and CAR scFv are used by different groups, which may influence the activity of anti-MSLN CAR T cells. Although targeting MSLN region I (like the SS1 scFv does) blocks the MSLN tumor-promoting interaction with MUC16 [14], a preclinical study suggested that MSLN region III is a better target as it mediated a stronger activation and cytotoxicity compared to CAR T cells targeting region I [20]. Genetic modification to express the CAR is obtained either transiently through mRNA expression or stably through viral vector transduction. Transient mRNA expression is considered safer as possible adverse effects are limited to the time of transient CAR expression and can therefore be better controlled. However, efficacy is also constrained by the transient CAR expression and recurrent injections may be needed to induce a sustained response. Reduction of possible adverse effects can be obtained also in stably transduced CAR T cells by including a suicide gene, as MSKCC has done (NCT02414269). Lymphodepletion prior to anti-MSLN cell therapy is used in the majority of trials and seems to aid CAR T cell expansion, however efficacy and persistence of anti-MSLN CAR T cells remains low and further research is needed to determine the antitumor benefit of its use. Different strategies have been implemented to resist PD-1 mediated exhaustion. The combination of anti-MSLN CAR T cell therapy with an anti-PD-1 CI is used in two trials and preliminary results from trial NCT02414269 shows promising synergy between the two therapies. Intrinsic mechanisms to avoid PD-1-derived exhaustion are being used in two trials: PD-1 DNR (NCT04577326) and secretion of PD-1 nanobodies (NCT04489862). The majority of trials use systemic infusion for the delivery of anti-MSLN CAR T cells and only 4 trials are testing local delivery, which could be an advancement towards more efficient tumor infiltration and homing of anti-MSLN CAR T cells.

It is too early to determine which anti-MSLN CAR T cell approach works best against MM. The majority of trials are in phase I or I/II with safety as their primary endpoint. The results so far are encouraging as all trials show manageable toxicity (mostly lower grade adverse effects and no ‘on target/off tumor’ toxicities), thereby confirming MSLN as a promising target for CAR T cell therapy against MM. Response rates have remained low. However, it is important to note that efficacy testing was not the primary endpoint of the trials listed and that CAR T cell dosage still needs to be optimized. Moreover, efforts are being made to increase anti-MSLN CAR T cell therapy efficacy, in terms of CAR design as well as enhancement of CAR T cell resistance to the TME immunosuppressive stimuli.

## Improving anti-Mesothelin CAR design

The identity of the intracellular costimulatory domain has an effect on CAR T cell phenotype and consequently on persistence and efficacy in vivo [46]. The costimulatory domains should therefore be carefully considered during CAR design, based on the tumor characteristics. Ideal features of anti-MSLN CAR T cells against MM include: a favorable safety profile, ability to infiltrate and home to the tumor, strong anti-tumor activity, resistance to TME-induced exhaustion, and long persistence.

The stimulatory domains CD28/CD3ζ and 4–1-BB/CD3ζ are widely used in CAR constructs as they were initially developed for CD19 CAR T cell therapy and have shown great success in leukemia and lymphoma [22]. They elicit different signaling strengths: CD28/CD3ζ CARs produce a strong, quick signal associated with an effector T cell-like phenotype and higher susceptibility to exhaustion and low persistence [47]. 4–1-BB/CD3ζ CARs have a weaker more prolonged signal associated with a memory T cell-like phenotype and more sustained anti-tumor activity in vivo [25, 48]. Several groups are exploring other costimulatory domains to improve potency, function and persistence (Fig. 2d).

A costimulatory domain used in anti-MSLN CAR T cells to increase their persistence and maintain a strong effector response is the Inducible T cell co-stimulator (ICOS) [49]. Third generation anti-MSLN CAR T cells carrying the anti-MSLN SS1 scFv and the combination of ICOS and 4–1-BB costimulatory domains were shown to produce enhanced anti-tumor effects and increased persistence in mouse models of ovarian and pancreatic cancers compared to second generation CD28 and 4–1-BB anti-MSLN CARs [50]. Moreover, ICOS and CD28 share the same signaling motif YMXM in their intracellular domains, with the difference being that X is asparagine (N) in CD28 and phenylalanine (F) in ICOS, thus promoting different downstream signaling cascades. Interestingly, Guedan et al. observed that changing the CD28 YMNM motif to YMFM in anti-MSLN CAR T cells produced stronger anti-tumor efficacy, increased persistence and avoided the development of the exhausted phenotype in a pancreatic cancer mouse model, compared to anti-MSLN CAR T cells carrying the routine CD28 motif [46].

Wang et al. at the University of Pennsylvania took an alternative CAR engineering approach and designed a CAR based on a natural multi-chain immunoreceptor comprising the killer immunoglobulin-like receptor (KIR) and DAP12, an ITAM containing molecule. KIR is naturally expressed by CD4+ and CD8+ T cells and binds DAP12 to stimulate cytotoxicity. They tested this design bound to an anti-MSLN SS1 scFv (SS1-KIRS2/DAP12 T cells) in xenograft models of MM and compared its function with second generation CD3ζ-based CAR T cells containing either CD28 or 4–1-BB as costimulatory domains. They found that SS1-KIRS2/DAP12 T cells had superior anti-tumor activity and showed strong cytotoxicity even in mesothelioma models resistant to CD3ζ-based immunotherapy. This success is likely due to more efficient maintenance of CAR expression on the T cell surface and improved effector function given by stimulation via KIR and DAP12 [51].

Choosing the right costimulatory domains is an essential but difficult task as CAR function depends on multiple exogenous factors such as antigen density, CAR affinity and TME immune characteristics [12]. Further studies examining the different costimulatory domains in correlation with MM biomarkers are required to determine which ones lead to a better clinical response.

## Overcoming tumor microenvironment barriers in anti-Mesothelin CAR T cell therapy

Clinical trials using anti-MSLN CAR T cell therapy have consistently produced a favorable safety profile but generally exhibit modest response rates. The TME plays a critical role in diminishing the efficacy of anti-MSLN CAR T cells in MM, so modulating their response to TME stimuli may increase their effectiveness (Fig. 2). The chronic inflammatory response to asbestos in the lungs and pleura contributes to a unique TME characterized by immunosuppressive T_regs_, M2 tumor-associated macrophages (TAM) and myeloid-derived suppressor cells. Their presence in the TME is associated with poor prognosis [28, 52, 53]. Moreover, MM is characterized by very poor infiltration of immune cells, and the intratumoral presence of CD8+ T cells is associated with a more favorable prognosis [54]. MM is surrounded by a strong physical barrier, the stroma, which plays a role in tumor progression, invasion and inhibition of anti-tumor immune responses. This barrier often prevents CAR T cells infused systemically from homing to the tumor, which is a critical step for the success of this type of therapy [55]. Moreover, cytokines and chemokines produced by cancer and stromal cells, such as TGF-β, IL-6 and CCL2, contribute to the recruitment and differentiation of immunosuppressive cells, which in turn further reduce CAR T cell potency in the tumor [28].

Strategies to increase anti-MSLN CAR T cells tumor infiltration are being tested in preclinical and clinical studies. One potential approach to overcome the physical barrier of the stroma is to perform regional delivery. When anti-MSLN CAR T cells were injected in the pleura or the peritoneum in preclinical studies, a higher success rate was observed than when they were infused intravenously. Intrapleural delivery also required a 30-fold lower dose of anti-MSLN CAR T cells for successful tumor eradication compared to intravenous injection [26]. Translated into the clinic, a lower CAR T cell dosage may help to avoid excessive release of cytokines and reduce production costs [37]. Consequently, locoregional anti-MSLN CAR T cell delivery is currently trialed in clinical studies (NCT03608618, NCT02414269, NCT03054298 and NCT04577326).

Moon et al. managed to aid trafficking to the tumor through the expression of the tumor homing chemokine receptor CCR2b on the surface of anti-MSLN CAR T cells which allows binding to the chemokine CCL2. The TME has abundant CCL2 as it is strongly secreted by MM cells. This approach showed increased tumor infiltration and a stronger anti-tumor activity in mice [56]. A similar strategy was used in a preclinical study combining MSLN CAR T cells with a modified oncolytic vaccinia virus. This virus induced tumor cells to produce larger amounts of CXCL11, a ligand of CXCR3, a chemokine receptor expressed at high level on central and effector memory T cells. This strategy elicited better tumor infiltration by anti-MSLN CAR T cells as well as enhanced anti-tumor activity [57].

Once CAR T cells successfully infiltrate the tumor mass, a second barrier emerges: the strong immunosuppressive TME in MM. There, CAR T cells encounter multiple inhibitory mechanisms that seem to be affected by histological subtype and previous anti-tumor therapies [58, 59]. On top of the T cell intrinsic inhibitory mechanisms exemplified by the expression of immune checkpoint molecules on their surface (e.g. PD-1) (Fig. 2c), anti-MSLN CAR T cells are also subjected to extrinsic suppressive features of the TME. These include soluble mediators TGF-β and prostaglandin E (PGE2), produced mostly by immunosuppressive M2 TAM and T_regs_ [60, 61], as well as adenosine, found at high levels as a result of hypoxia and expression of ectoenzymes CD39 and CD73 [62] (Fig. 2b). The PD-1/PD-L1 axis as well as TGF-β signaling have also been shown to convert T_h1_ cells (responsible for immune surveillance) to T_regs_ [63, 64].

Many groups are working on strategies to promote CAR T cell resistance against these inhibitory signals. Adding inhibitors of immune checkpoints to reinvigorate exhausted T cells is a good example (pembrolizumab in NCT02414269 and NCT03907852). However, the efficacy of CI can be limited by inefficient tumor infiltration and short half-life, so another option is to genetically armor CAR T cells against suppressive stimuli.

Anti-MSLN CAR T cells have been engineered to achieve intrinsic PD-1 checkpoint blockade by different methods. One method involves the knock out of PD-1 via CRISPR/Cas9 [65]. Cherkassky et al. added a PD-1 DNR in anti-MSLN CAR T cells which rescued their anti-tumor function in a MM mouse model [66, 67]. This strategy is used also in the ongoing phase I trial NCT04577326. Liu et al. engineered a PD-1/CD28 switch receptor, which carries the truncated extracellular domain of PD-1 and the transmembrane and cytoplasmic signaling domains of CD28, thus mediating CAR T cell activation instead of exhaustion [68]. Overall, intrinsic PD-1 blockade has been shown to increase CAR T cells anti-tumor activity in preclinical studies and it is hoped it will also enhance their functional persistence in solid tumors.

CRISPR/Cas9 technology has also been used to knock out the TGF-β receptor II (TGFBR2) and render anti-MSLN CAR T cells resistant to the adverse effect of TGF-β signaling, thus rescuing their proliferation potential, potency and anti-tumor activity in MM mouse models [69]. Alternatively, anti-MSLN CAR T cells can be used in combination with oncolytic viruses targeting TGF-β-expressing cancer cells. This strategy produced a stronger anti-tumor response compared to monotherapy in mouse models of breast cancer [70]. Interestingly, in an effort to overcome impaired anti-tumor immunity in CD8+ anti-MSLN CAR T cells, Riese et al. deleted diacylglycerol kinase, whose activity limits the strength of TCR signaling. They observed enhanced potency and anti-tumor activity against MM cell lines, and they also showed a reduced sensitivity to TGF-β [71].

In in vitro experiments, the inhibitory effect of adenosine was reduced by targeting the adenosine 2A receptor (A_2A_R) whose expression is upregulated after activation in T cells. A_2A_R expression was knocked down via shRNA in anti-MSLN CAR T cells and prompted enhanced proliferation, cytokine production and cytotoxicity. Interestingly, pharmacological targeting of A_2A_R via antibody did not elicit the same improved cytotoxicity function as obtained by A_2A_R knock down by shRNA [72].

Prostaglandin E (PGE2) and adenosine can also mediate immunosuppression through the activation of Protein Kinase A (PKA). Once activated, PKA binds to the membrane protein ezrin in the immune synapse causing the inhibition of TCR activation. Newick et al. created anti-MSLN CAR T cells that express the ‘regulatory subunit I anchoring disruptor’ (RIAD) which inhibits the association of PKA with ezrin. Blocking PKA localization to the immune synapse led to increased anti-MSLN CAR T cell migration to the tumor as well as enhanced anti-tumor efficacy in a MM mouse model [73].

In order to increase the efficacy of anti-MSLN CAR T cells, they were combined with oncolytic adenoviruses expressing the cytokines TNF-α and IL-2, which mediate T cell activation and proliferation. This combination showed increased anti-tumor activity compared to monotherapy in mouse models of PDAC. Moreover, the oncolytic virus addition increased T cell infiltration and altered the host immune status. The PDAC tumors revealed M1 polarization of macrophages and increased dendritic cells maturation, thus implying that this combination may be used to revert TME immunosuppression [74].

A recently developed approach is the genetic modification of macrophages with CARs. The human macrophage THP-1 cell line was stably transduced with an anti-MSLN CAR containing the CD3ζ signaling domain (CAR-M). Antigen-dependent phagocytic activity was shown in vitro against MM cell lines. Further in vivo experiments were carried out using primary monocyte-derived CAR-M targeting HER2 in an ovarian cancer xenograft mode. The positive results obtained make this approach a possible option in MM too, using anti-MSLN CAR-M. Anti-HER2 CAR-M elicited decreased tumor burden and prolonged overall survival. Moreover, they had an effect on the TME and prompted the conversion of bystander immunosuppressive M2 macrophages to pro-inflammatory M1. Anti-HER2 CAR-M were resistant to the suppressive TME, recruited adaptive immunity and enhanced the anti-tumor activity of T cells [75].

Other strategies to enhance CAR T cell tumor infiltration, efficacy and persistence were reviewed a few years ago [12], but are not currently being tested clinically with anti-MSLN CAR T cells.

The immunosuppressive TME is a serious obstacle towards effective anti-MSLN CAR T cell therapy in MM but much progress has been made in recent years. Approaches include the exploration of T cell infiltration and exhaustion signaling pathways and their manipulation to enhance the efficacy of CAR T cell therapy. Once these strategies are successfully translated into the clinic, there is hope that anti-MSLN CAR T cell therapy will improve MM patient outcomes.

## Other T cell therapy targets in mesothelioma

Two other TAA for T cell therapy against MM have been tested in clinical trials: Fibroblast Activation Protein (FAP) and Wilms tumor 1 (WT1). FAP is highly expressed on cancer-associated stromal cells and is also detected in the different histological subtypes of MM. Targeting FAP has therefore the potential to increase tumor infiltration as well as anti-tumor activity by disrupting the extracellular matrix components [76]. Three patients were treated in a phase I trial testing the safety anti-FAP CD8+ CAR T cells administered intrapleurally (NCT01722149) and no serious toxicities were observed [77, 78]. WT1 is an oncogenic zinc-finger transcription factor expressed intracellularly in the majority of MM cases. To target this intracellular TAA, an engineered TCR instead of a CAR is needed on the T cell surface. An ongoing phase I/II trial is testing anti-WT1 TCR T cells in MM patients expressing WT1 and human leukocyte antigen (HLA)-A*0201. Central memory and naïve CD8+ T cells were selected for TCR transduction in the hope to increase persistence and efficacy of this therapy (NCT02408016).

CAR T cell therapy strategies targeting other TAA in MM are still in the preclinical phase but have shown promise. The targeted TAA include: MET [79], Pan-ErbB T4 [80], 5 T4 [81] and Chondroitin sulfate proteoglycan 4 (CSPG4) [82]. There are also CAR T cell therapies targeting the tumor vasculature which have shown encouraging results in mouse studies in other cancer types and may be of potential benefit in MM [83].

## Conclusions

MM is a treatment-resistant tumor with few approved treatment options, however novel immunotherapeutic approaches have renewed hope for improved clinical outcomes, especially anti-MSLN CAR T cell therapy. The clinical trial landscape using this therapy in MM shows the variety of strategies adopted in terms of CAR constructs, transduction methods, combination therapy, and delivery methods. Despite the limited response rates observed in trials against MM so far, anti-MSLN CAR T cell therapy represents a promising new treatment modality. Firstly, MSLN has been shown to be a specific and safe target in MM; and secondly, the ongoing research on CAR design and efficacy enhancement of anti- MSLN CAR T cells as well as their combination with CI and oncolytic viruses has increased tumor infiltration, efficacy and persistence. This progress in overcoming the barriers caused by the TME allows for an optimistic perspective once these anti-MSLN CAR T cell therapy augmentations are successfully translated into the clinic.

## Data Availability

The clinical trials using anti-mesothelin CAR therapy were sourced from the website Clinicaltrials.gov, accessed in October 2020. Figures were created with BioRender.com (2020).

## Abbreviations

CAR: Chimeric antigen receptor
CI: Checkpoint inhibitors
CTLA-4: Cytotoxic T-lymphocyte-associated protein 4
DNR: Dominant negative receptor
FAP: Fibroblast Activation Protein
ICOS: Inducible T cell co-stimulator
ITAM: Immunoreceptor tyrosine-based activation motif
KIR: Killer immunoglobulin-like receptor
KO: Knock out
MM: Malignant mesothelioma
MPF: Megakaryocyte potentiating factor
MPM: Malignant pleural mesothelioma
MSKCC: Memorial Sloane Kettering Cancer Center
MSLN: Mesothelin
MUC16: Mucin 16
NSCLC: Non-small cell lung cancer
PBMC: Peripheral blood mononuclear cells
PD: Progressive disease
PD-1: Programmed cell death protein-1
PD-L1: Programmed cell death ligand-1
PDAC: Pancreatic ductal adenocarcinoma
PKA: Protein Kinase A
RIAD: Regulatory subunit I anchoring disruptor
scFv: single-chain variable fragment
SD: Stable disease
SMRP: Soluble mesothelin-related peptide
TAA: Tumor-associated antigen
TAM: Tumor-associated macrophages
TCR: T cell receptor
TME: Tumor microenvironment
T_regs_: Regulatory T cells
WT1: Wilms tumor 1
